# Strangulated inguinal hernia due to an omental band adhesion within the hernial sac: a case report

**DOI:** 10.1186/1757-1626-2-21

**Published:** 2009-01-07

**Authors:** Senthil Nachimuthu, Szabolcs Gergely

**Affiliations:** 1 Department of Surgery, Hinchingbrooke Hospital, Hinchingbrooke Healthcare NHS Trust, Huntingdon, Cambridgeshire, UK

## Abstract

**Introduction:**

Strangulated Inguinal hernia is one of the most common surgical emergencies dealt with by surgeons worldwide. Usually the narrow internal inguinal ring or the external inguinal ring is the site of constriction of the viscus, which forms the content of the hernia resulting in strangulation. We report a rare case of strangulated inguinal hernia where the constricting element is not the internal or external inguinal ring, but an omental band adhesion causing closed loop small bowel obstruction and gangrene within the hernial sac in the inguinal canal.

**Case Report:**

A 56-year-old Caucasian gentleman presented to us with a 6 hours history of non-reducible tender lump in his right groin. His groin was explored urgently under general anaesthesia and was found to have an omental band adhesion causing closed loop small bowel obstruction with gangrene within the hernial sac in the inguinal canal with a wide internal inguinal ring. Gangrenous small bowel was resected and primary anastomosis was performed through the same inguinal incision.

**Conclusion:**

Strangulation of the inguinal hernial content is usually due to the tight constriction at the level of internal inguinal ring or at external inguinal ring. Uncommonly strangulation of the contents can occur due to other causes like omental band adhesion. Anyone presenting with clinical features of strangulated inguinal hernia with small bowel obstruction mandates prompt exploration of the inguinal canal. Although it may not change the treatment approach, one should be aware about this special entity. Resection of the gangrenous small bowel and primary anastomosis can be safely performed through the same inguinal incision.

## Introduction

Strangulated Inguinal hernia is one of the most common surgical emergencies dealt with by surgeons worldwide. It is the most common cause of intestinal obstruction in all age groups. Usually the narrow internal inguinal ring or the external inguinal ring is the site of constriction of the viscus, which forms the content of the hernia resulting in strangulation. Herewith we present an extremely rare case of strangulated inguinal hernia where the constricting element is not the internal or external inguinal ring, but an omental band adhesion causing closed loop small bowel obstruction and gangrene within the hernial sac in the inguinal canal. To the best of our knowledge, this is the first ever case reported in the world literature of such type.

## Case Report

**Figure 1:**
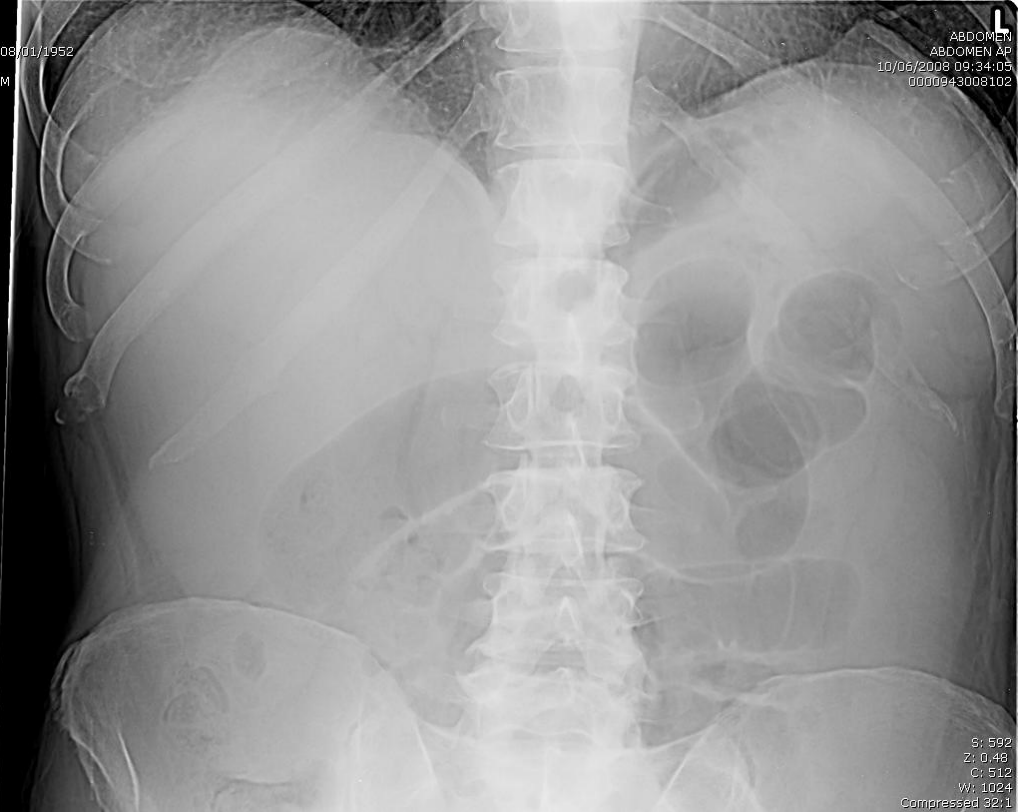
**Plain abdominal x-ray**. Distended small bowel loops consistent with small bowel obstruction.

**Figure 2:**
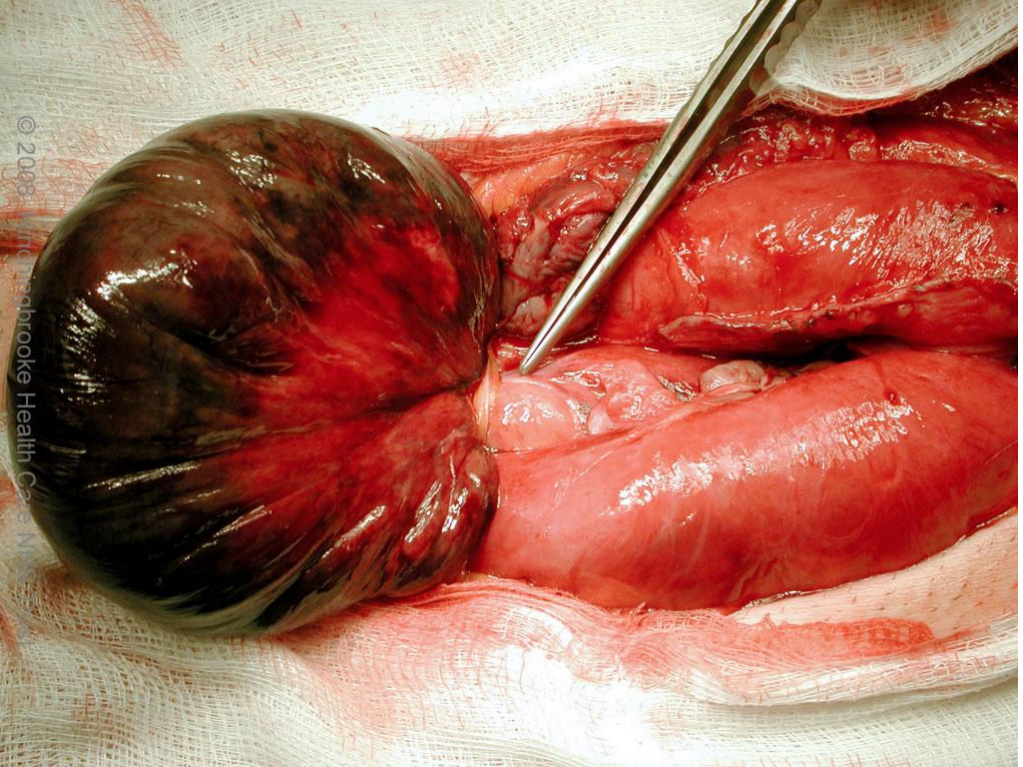
**Intra-operative photograph**. Gangrenous small bowel with closed loop obstruction caused by an omental band adhesion.

A 56 year old Caucasian gentleman presented with a six hour history of a non-reducible tender lump in his right inguinal region. He was known to have bilateral inguinal hernia for the past three months and was waiting for an elective repair. His past medical history includes systemic hypertension for which he is on anti-hypertensive medications. There is no history of previous abdominal operations or any episodes of abdominal sepsis. On clinical assessment, his temperature was 37.5 degree Celsius, heart rate was 90 beats per minute and blood pressure was 130/90 mm of Hg. Local examination revealed a 15 cm × 7 cm sized non-reducible tender swelling in his right inguinal region with no cough impulse. Contralateral side revealed a non-tender, reducible inguinal hernia. Examination of the abdomen revealed mild distension. Blood biochemistry results were as follows: Haemoglobin 14.5 gm/dl, White cell count 25,000 cells/mm[3], Neutrophil count 22,500 cells/mm[3], Urea 4.0 mmol/L, Creatinine 68 mmol/L, Potassium 4.0 mmol/L, Sodium 135 mmol/L, C – Reactive protein 67 mg/l. Plain abdominal x-ray showed multiple loops of distended small bowel seen centrally in the abdomen consistent with distal small bowel obstruction. (Figure 1) He was taken to the operation theatre urgently with a diagnosis of strangulated inguinal hernia and the right inguinal region was explored under general anaesthesia. Gangrenous small bowel of 20 cm with closed loop obstruction caused by a single omental band adhesion was noted. (Figure 2) The neck of the hernial sac at the level of the internal inguinal ring as such was found to be very wide. The omental band adhesion was divided and the gangrenous small bowel was resected and primary stapled anastomosis was performed through the same inguinal incision. The widened internal inguinal ring was narrowed and the posterior wall of the inguinal canal was repaired with sutures rather than mesh due to the presence of infection. The patient made an uneventful post-operative recovery and was discharged home on the third post-operative day. Histology of the resected specimen was reported as transmural infarction of the small bowel with viable resection margins and no evidence of intravascular thrombosis or vasculitis. He was followed up in the out-patient clinic four weeks later and found to have no problems and has been booked for an elective hernia repair on the contralateral side.

## Discussion

Strangulated Inguinal hernia is one of the common surgical emergencies dealt with by surgeons worldwide and it is one of the most common causes of intestinal obstruction in all age groups[1]. There are various intra-abdominal conditions, which can present within the inguinal hernial sac and clinically mimic as strangulated inguinal hernia. Of note, pathologies like obstructed and perforated sigmoid tumours have been reported in the literature on many occasions manifesting as strangulated inguinal hernia[2-4]. Sigmoid diverticular abscess presenting as strangulated inguinal hernia has also been reported[5,6]. Other conditions like acute pancreatitis[7] and bilioma secondary to spontaneous rupture of biliary system[8] have also been reported as manifesting clinically as strangulated inguinal hernia. Omental band adhesion causing small bowel obstruction and gangrene within the inguinal hernial sac and clinically presenting as strangulated inguinal hernia is one another type of this special clinical entity. To the best of our knowledge, this is the first ever case reported in the world literature of such type. Omental band adhesion causing acute small bowel obstruction inside the general peritoneal cavity is a very well recognized entity. The aetiology of the omental band formation could be congenital, adhesions secondary to previous operations, inflammation etc., The most likely explanation in our case, though not impossible in his age, would be congenital as he did not undergo any abdominal operations in the past and there was no history of any inflammatory abdominal conditions. Most of the other pathological conditions develop inside the general peritoneal cavity and then track along the normal anatomical pathways and enter the inguinal hernial sac. In women, sigmoid diverticular abscess can track along the round ligament and enter the inguinal canal mimicking as strangulated inguinal hernia[6]. Bilious fluid secondary to peptic ulcer perforation and spontaneous biliary perforation[8] and pancreatic fluid collections secondary to pancreatitis enter the inguinal hernial sac through the internal ring. Hence one should be cautious in making a decision to explore the inguinal canal in such situations, as most of the times; the situation resolves when the primary pathology settles down. Nevertheless, anyone presenting with clinical features of strangulated inguinal hernia with small bowel obstruction mandates prompt surgical exploration of the inguinal canal as was done in our case. Most of the times, small bowel resection can be performed safely through the inguinal incision if the viability of the necessary extent of the bowel could be assessed in a satisfactory manner.

## Conclusion

Strangulation of the inguinal hernial content is usually due to the tight constriction at the level of internal inguinal ring or at external inguinal ring. Various other intra-abdominal conditions can present inside the inguinal hernial sac and mimic as strangulated inguinal hernia. In such cases, one can safely manage the situation by treating the primary condition without exploring the inguinal canal and of course, with caution. Uncommonly strangulation of the contents can occur due to other causes like omental band adhesion within the hernial sac. Anyone presenting with clinical features of strangulated inguinal hernia with small bowel obstruction mandates prompt surgical exploration of the inguinal canal. Although it may not change the treatment approach, one should be aware about this special entity.

## Consent

"Written informed consent was obtained from the patient for publication of this case report and accompanying images. A copy of the written consent is available for review by the Editor-in-Chief of this journal."

## Competing interests

The authors declare that they have no competing interests.

## Authors' contributions

SN performed the operation, designed the case report, drafted the manuscript and conducted critical literature review. SG critically analysed and revised the manuscript for important intellectual content.
